# Soil water solutes reduce the critical micelle concentration of quaternary ammonium compounds

**DOI:** 10.1007/s11356-020-10188-2

**Published:** 2020-08-12

**Authors:** Ines Mulder, Malte Schmittdiel, Henning Frei, Laura Hofmann, Dennis Gerbig, Jan Siemens

**Affiliations:** 1 Institute of Soil Science and Soil Conservation, iFZ Research Centre for BioSystems, Land Use and Nutrition, Justus–Liebig University Giessen, Heinrich-Buff-Ring 26-32, 35392 Giessen, Germany; 2 Institute of Organic Chemistry, Justus–Liebig University, Heinrich-Buff-Ring 17, 35392 Giessen, Germany

**Keywords:** Fluorescence, Critical micelle concentration, Quaternary alkyl ammonium compound, Dissolved organic matter

## Abstract

Quaternary alkyl ammonium compounds (QAACs) are produced in large quantities for use as surfactants and disinfectants and also found in soils, sediments, and surface waters, where they are potentially involved in the selection of antibiotic resistance genes. Micelle formation influences fate and effects of QAACs. The critical micelle concentration (CMC) of six homologs of benzylalkylammonium chlorides (BAC) was determined in deionized water, 0.01 M CaCl_2_ solution, and aqueous soil extracts, using both spectrofluorometric and tensiometric methods. Additionally, eight organic model compounds were employed at concentrations of 15 mg C L^−1^ as background solutes in order to test the effect of dissolved organic carbon (DOC) on CMCs. Results found CMCs decreased with an increasing length of the alkyl chain from 188 mM for BAC-C8 to 0.1 mM for BAC-C18. Both methods yielded similar results for measurements in water and CaCl_2_ solution; however, the spectrofluorescence method did not work for soil extracts due to fluorescence quenching phenomena. In soil extracts, CMCs of BAC-C12 were reduced below 3.7 mM, while the CMC reduction in soil extracts was less pronounced for BAC-C16. Besides ionic strength, molecular structures of BACs and dissolved organic compounds also affected the CMC. The number of carboxyl groups and small molecular weights of the DOC model compounds reduced the CMCs of BAC-C12 and BAC-C16 at pH 6. This study highlights that CMCs can be surpassed in soil solution, pore waters of sediments, or other natural waters even at (small) concentrations of QAACs typically found in the environment.

**Electronic supplementary material:**

The online version of this article (10.1007/s11356-020-10188-2) contains supplementary material, which is available to authorized users.

## Introduction

Quaternary alkyl ammonium compounds (QAACs) are a large group of structurally varying compounds that are frequently employed as surfactants and disinfectants in a vast spectrum of applications, from oil drilling fluids, to surface cleaning agents to cosmetic hair formulations. It has been noted that after their use, QAACs accumulate in sewage sludge during wastewater treatment (Martínez-Carballo et al. 2007; Zhang et al. 2015; Östman et al. 2017), and also in estuarine sediments (Lara-Martín et al. 2010; Li and Brownawell 2010). The environmental fate of QAACs is currently not well understood. Several substances in this class belong to the so-called high production volume chemicals (Organization for Economic Co-operation and Development 2009) and have an annual production rate as high as 700.000 t (Jennings et al. 2015). Known pathways for QAAC agricultural contamination of soils and sediments include the application of wastewater/sewage sludge, as well as the application with pesticide formulations, and manures (Mulder et al. 2018). The correlation of QAACs in the environment with antibiotic resistance gene development has raised concerns about their environmental fate and effects (Jechalke et al. 2014; Jennings et al. 2015).

At the same time, surfactants containing QAACs are proposed for the remediation of polycyclic aromatic hydrocarbons (PAH) contaminated soils or the remediation of water repellant soils (Gitipour et al. 2020; Ashraf et al. 2020; Ogunmokun et al. 2020), which highlights the need of an in-depth understanding of QAAC fate and effects in soils. In remediation technology, excavated soils that were polluted by an oil spill with hydrophobic organic pollutants undergo a surfactant-enhanced washing procedure, where, at surfactant concentrations below the critical micelle concentration (CMC), surfactant molecules are adsorbed on the contaminant surfaces and repulsion between head group and soil particles mobilizes the contaminant; this mechanism is also termed soil roll-up (Deshpande et al. 1999). A second mechanism for contaminant removal comes into effect above the CMC, when the hydrophobic organic contaminants are solubilized within the formed micelles (Mao et al. 2015). In soils, the repulsion of primarily negatively charged soil particles would attract the positively charged head groups of QAACs and this second mechanism at surpassed CMC becomes the dominant mechanism. Ranjan et al. (2006) reported that a combined in situ treatment of a QAAC and electrokinetics retarded the movement of hydrocarbon contaminants in soils.

One important physicochemical parameter for the characterization of surfactants is thus their critical micelle concentration (CMC). Below the CMC, surfactant molecules are distributed evenly as monomers throughout the solution medium. Once the CMC is reached, these monomers start to associate to form spherical micelles, which profoundly affects how they behave in solution and affects characteristics such as their solubilizing effect towards other (organic) solutes, and their interaction with surfaces (Kralchevsky et al. 2002).

Although the CMC by definition is not a precise value, it rather marks the turning point in the transition from unassociated monomers to micelles. This point serves as an important characteristic parameter for assessing the behavior of QAACs in solution and their sorption on surfaces. Micelle formation is accompanied by a change in physicochemical properties, such as osmotic pressure, solubilization, surface tension, electric conductivity, self-diffusion, turbidity, or magnetic resonance (Tadros 2005). In an aqueous polar environment, micelles will configure itself to where hydrophobic alkyl chains spherically face the middle, shielded by polar hydrophilic head groups (here the charged nitrogen atom), forming an energetically favorable aggregation. Over a wide concentration range above the CMC, micelles can be thought of as liquid hydrocarbon droplets covered with polar head groups, by which they interact with water molecules (Tadros 2005). Correspondingly, hydrophobic tail and hydrophilic head groups would form inverse micelles in apolar solvents. Also, mixed micelles can be composed of different surfactant types, such as cationic and nonionic, or of surfactants with the same head group, e.g., QAACs, but with different chain lengths. In the latter case, no net interaction will occur, i.e., the CMC of such a mixed micelle should correspond to the average CMC of the single components (Tadros 2005). However, this “mixed surfactant solution”—CMC—needs to be considered carefully as micelles in mixed binary surfactant systems were found to form asynchronously with the low CMC component aggregating first (Cui et al. 2010).

Other parameters, such as octanol/water partitioning coefficient or p*K*_a_ values, are not applicable for QAACs, so CMC is a key parameter in assessing environmental fate and effect. In a study by Ismail et al. (2010), CMCs were correlated to the *K*_F_ Freundlich adsorption isotherm parameter. The authors argued that as the CMCs represent both hydrophobic and ionic properties of QACs, they are an effective descriptor of QAAC partitioning on biosolids.

There are various methods for determining CMCs of surfactants. Literature reports several values for CMCs of benzylalkylammonium compounds (BACs) as a selected group of structurally similar QAACs. These studies are typically concerned with thermodynamic observations (del Castillo et al. 2000; Asakawa et al. 2001; González-Pérez et al. 2001; Marcotte et al. 2005; Avranas and Gernátová 2007; Zdziennicka et al. 2012). Itoh et al. (2005), while interested in photodegradation of micelles, demonstrated that the CMC decreased in the order BAC-C8 < BAC-C12 < BAC-16, as expected from the increased hydrophobic interaction with increasing alkyl chain length. Gonzáles-Pérez et al. (2001) showed that CMCs are temperature-dependent and that several BAC homologs show a slight CMC minimum at a temperature of 35 °C. The abovementioned studies all have in common that CMCs are determined in aqueous media, mostly DI water with the methods for the determination of CMCs ranging from conductometry to densitometry, calorimetry, UV/Vis spectrometry, and fluorescence quenching methods.

Within the group of QAACs, BACs belong to the most frequently employed compounds with sediment concentrations in the μg g^−1^ range and sewage sludge concentrations even in the mg g^−1^ range (Zhang et al. 2015; Mulder et al. 2018). The structure of BACs consists of the central nitrogen substituted by two methyl groups, one benzyl ring and an alkyl chain of varying chain length. The permanent positive charge on the nitrogen atom conveys a hydrophilic character to the molecule, whereas the alkyl chain is responsible for its hydrophobicity. In soils, BACs will trend to be adsorbed to negatively charged surfaces of soil particles but to date, little is known about the fate of BACs in soils (Mulder et al. 2018). Overall, the molecular structure is responsible for the BAC amphiphilic character, which determines their application as surfactants. Several BAC compounds also possess good disinfectant properties, which is utilized for example in corrosion inhibitors and cleaning agents used in animal husbandry (Mulder et al. 2018).

Table 1 shows molecular properties and literature values for six BAC homologs, including their CMCs. Published data summarized in Table 1 imply that CMCs will unlikely be surpassed at the predicted environmental concentration of QAACs, which are in the range of 0.01–4.9 mg L^−1^ for river water and wastewater (Mulder et al. 2018). However, soil solutions, groundwater, and surface waters contain natural dissolved organic matter (DOM) as well as inorganic solutes and are thus markedly different from the aqueous medium in which CMCs were typically determined in other studies. Factors that can affect the CMC of surfactants are the structure of the surfactant, the presence of electrolyte or organic compounds, and the temperature of the solution (Rosen and Liu 1996). We expected that with increasing ionic strength of the background electrolyte, CMCs should decrease as the elevated polarity difference between medium and BAC tails would favor their micellization, also known as the counter ion effect (Ishiguro and Koopal 2016). Therefore, we hypothesized that CMCs must be lower in soil solutions, compared with DI water, due to the elevated concentration of solutes and greater ionic strength. Our second hypothesis was that the organic molecules, as contained in dissolved organic matter (DOM), plus inorganic solutes, will cause a further reduction of CMC.

**Table 1:** Quaternary benzylalkylammonium compounds employed in this study and their physicochemical properties. The CMC values are extracted from literature and their superscripted characters correspond to the following references: a = González-Pérez et al. 2001, b = Marcotte et al. 2005, c = Zdziennicka et al. 2012, d = Ismail et al. 2010, e = Lemić et al. 2005, and f = Asakawa et al. 2001

Compound name	Acronym	CAS#	Molecular structure	Mol mass HPVC		mp (°C)	CMC (mM)*
Benzylalkyldimethylethylammonium compounds	BACs	8001-54-5	Variable, with C8-C18 alkyl chain	Variable	No	n/a	n/a
Octylbenzyldimethylammonium chloride	BAC-C8	959-55-7	CH3(CH2)7N(Cl)(CH3)2CH2C6H5	283	No	n/a	n/a
Decylbenzyldimethylammonium chloride	BAC-C10	965-32-2	CH3(CH2)9N(Cl)(CH3)2CH2C6H5	311	No	n/a	38.3–38.7^a^
Dodecylbenzyldimethylammonium chloride^§^	BAC-C12	139-07-1	CH3(CH2)11N(Cl)(CH3)2CH2C6H5	339	Yes	42	6.2*10^−3^–3.8^bcd^
Tetradecylbenzyldimethylammonium chloride^§^	BAC-C14	139-08-2	CH3(CH2)13N(Cl)(CH3)2CH2C6H5	368	Yes	63	1.99–2.16^aef^
Hexadecylbenzyldimethylammonium chloride^§^	BAC-C16	122-18-9	CH3(CH2)15N(Cl)(CH3)2CH2C6H5	396	Yes	59	0.004–0.6^abd^
Octadecylbenzyldimethylammonium chloride^§^	BAC-C18	122-19-0	CH3(CH2)17N(Cl)(CH3)2CH2C6H5	424	Yes	57	0.86^e^

^*^References see figure capture

^§^High production volume chemicals (according to OECD)

The overarching aim of our work was to systematically enlarge the CMC database of BACs to improve our understanding of the effects of BACs in the soil and sedimentary environment and to assess the associated risks.

## Experimental

### Chemicals

The following chemicals were used without further purification: benzyldimethyloctylammonium chloride (BAC-C8), purity > 96% (CAS# 959-55-7, Sigma-Aldrich); benzyldimethyldecylammonium chloride (BAC-C10), purity > 97% (CAS# 965-32-2, Sigma-Aldrich); benzyldimethyldodecylammonium chloride dihydrate (BAC-C12), purity > 98% (CAS# 139-07-1, Tokyo Chemical Industry); benzyldimethyltetradecylammonium chloride hydrate (BAC-C14), purity > 98% (CAS# 139-08-2, Tokyo Chemical Industry); benzylcetyldimethylammonium chloride hydrate (BAC-C16), purity > 95% (CAS# 122-18-9, Tokyo Chemical Industry); benzyldimethylstearylammonium chloride hydrate (BAC-C18), purity > 98% (CAS# 122-19-0, Tokyo Chemical Industry); calcium chloride dehydrate p.A. (CAS# 10035-04-8, Merck); potassium chloride p.A. (CAS#7447-40-7, VWR); sodium chloride p.A. (CAS# 7647-14-5, Carl Roth); methanol HiPerSolv for chromatography (CAS#67-56-1, VWR); pyrene, purity 98% (CAS#129-00-0, Alfa Aeser); acetic acid (CAS#64-19-7, Acros Organics); oxalic acid dihydrate (CAS#6153-56-6, Carl Roth); maleic acid (CAS#110-16-7, Carl Roth); D-(+)-galacturonic acid monohydrate (CAS#91510-62-2, Sigma-Aldrich); oxaloacetic acid (CAS#328-42-7, Carl Roth); citric acid dihydrate (CAS#6132-04-2, Merck); D-(+)-glucose monohydrate (CAS#50-99-7, Merck); and hydroquinone (CAS#123-31-9, Carl Roth). All aqueous solutions were prepared using deionized (DI) water prepared by reverse osmosis. A 0.01-M CaCl_2_ solution was prepared to mimic electrolyte content of soil solution as typically employed for, e.g., soil pH determination (Thomas 1996).

### Soil samples

Four different (top) soil samples were used in this study in order to reflect diverse genetic and land use conditions, and a variable chemical composition.

Soil samples of an agriculturally used fluvisol (WRB 2005) were taken from the research farm “Weilburger Grenze” (WG) of the Justus Liebig University, located at the NW outskirts of Giessen. The site is situated 158 m above mean sea level and is characterized by an annual average rainfall of 650 mm and average annual air temperature of 9 °C. Reported soil texture on the research farm is silty clay with a clay content of 28–33%. This sample was used to prepare soil extracts WG1 and WG2.

Sample OH was from an agriculturally used luvisol taken from another research farm of the Justus Liebig University of Giessen, the “Oberer Hardthof”. Here, the site is located 200 m above mean sea level, with comparable average rainfall and temperature and a sandy loam texture.

Sample ISR is an arenosol taken in the interdune in the area of Nizzana-South, North West Negev, Israel, and located 190 m above mean sea level. The mean annual temperature of this area is 19.2 °C and average rainfall is 87 mm.

The fourth sample AP was taken at a forest site called Achenpass and located at the Swabian-Bavarian foothills of the Alps 1081 m above mean sea level. This soil is a rendzic leptic skeletic dolomitic phaeozem, and characterized by an organic matter content of between 47 and 60%. Average annual rainfall there is 2037 mm and temperature is 6.9 °C.

### Preparation and characterization of aqueous soil extract

Except for the soil extract WG1, which was prepared from a fresh field sample, all soil extracts were prepared from air-dried samples by shaking a 1:2.5 soil to DI-water suspension in centrifuge bottles for 24 h at 200 rotations per minute (rpm) on an orbital shaker (Swip KS-10, Edmund Bühler, Bodelshausen, Germany). Suspensions were subsequently centrifuged twice for 30 min at 1500 and 2400 g in a Rotanta 460 R centrifuge (Hettich, Tuttlingen, Germany). The slightly turbid supernatant was carefully decanted into a glass funnel equipped with a phosphate-free folded filter (MN 619 G1/4185 mm, Macherey-Nagel, Düren, Germany) to remove remaining fine precipitates from the supernatant and additionally filtered with a 0.45-μm filter. The resulting clear soil extract was homogenized by mixing in a 2000-mL volumetric glass flask by hand. For storage, the extract was split into four aliquots. All extracts were degassed by bubbling argon through the liquid for several minutes. The remaining headspace was flooded with argon. The soil extracts were stored at 4 °C in the refrigerator. After each use, the argon purging step was repeated in order to minimize microbial activity during storage.

The following parameters for each extract were determined: electrical conductivity (conductivity meter LF 95, WTW, Weilheim, Germany), pH (pH-meter handylab 2 equipped with a blue line pH 28 electrode, Schott Instruments, Weilheim, Germany), total organic carbon (TOC) as non-purgeable organic carbon (NPOC), and total organic nitrogen (as total nitrogen bound, TNb) using a TOC/TNb-analyzer (Vario-TOC analyzer, Elementar, Langenselbold, Germany), elements Al, Ca, Fe, K, Mg, Mn, Na, P, Pb, S, Cu, and Cr by inductively coupled plasma optical emission spectroscopy (ICP-OES spectrometer 720-ES, Varian, Palo Alto, USA) and ions Fl^−^, Cl^−^, NO_2_^−^, Br^−^, NO_3_^−^, SO_4_^2−^, and PO_4_^3−^ via ion exchange chromatography (ICS-2000, with AG 18 2 mm and AS 18 2 mm columns, Dionex, Sunnyvale, USA).

### CMC determinations with spectrofluorometry using the pyrene I/III-peak-ratio method

Pyrene is a hydrophobic polycyclic aromatic compound that is barely soluble in water. It tends to diffuse into the inner hydrophobic parts of micelles, in order to reduce interfaces with the polar medium, if present. The fluorescence properties of pyrene are expressed as relative intensities at fluorescence emission wavelengths near 373 nm and 384 nm of peak I and peak III, respectively, depending on the polarity of the surrounding medium. Pyrene can thus serve as a sensor for the detection of micelle formation. The I/III-peak-ratio of pyrene is subject to a sigmoidal decline in the concentration range in which micelles begin to form. The method is well documented and was earlier used to determine CMCs of a BAC homolog (Brito and Vaz 1986; Dominguez et al. 1997; Aguiar et al. 2003).

The pyrene fluorescence spectra were recorded on a FP8300 spectrofluorometer (JASCO, Tokyo, Japan) with an excitation wavelength of 334 nm (Dominguez et al. 1997), an excitation bandwidth of 5 nm, and an emission bandwidth of 2.5 nm, in the range from 350 to 450 nm. A detailed measurement report is provided in the supplementary information (SI).

For each measurement, the corresponding BAC concentration was adjusted in a high precision cell made of quartz SUPRASIL (Hellma Analytics, Müllheim, Germany). The background medium (DI water, 0.01 M CaCl_2_, quantity soil extract WG1) was added first, then BAC stock solution was added to the respective medium until a total volume of 3 mL was reached. Thirdly, 50 μL of a 25-mg L^—1^ pyrene solution in methanol was added. Finally, the cuvette was thoroughly shaken. After each measurement, the cuvette was rinsed three times with isopropanol, or, if high electrolyte concentrations were employed, with DI water and dried under vacuum.

### CMC determinations with tensiometry

The surface tension of a surfactant solution decreases with increasing surfactant concentration until the CMC is reached. As micelles begin to form, this decrease stops to continue and the surface tension will remain more or less constant above the CMC (Kronberg et al. 2014). In order to replicate experimental results obtained by spectrofluorometry with an independent method, the tensiometric Du Noüy ring method was selected (Noüy and Lecomte 1925). A LAUDA TD 2 (LAUDA Dr. R. Wobser GmbH, Lauda-Königshofen, Germany) tensiometer was used to determine the maximum force needed to pull a platinum ring at the meniscus of a 10-mL BAC solution, until a standard deviation smaller than 0.01 mN m^−1^ was achieved, five consecutive times. The reported surface tension value is a mean value of the last five measurements. Tensiometry was used to determine CMCs of BAC homologs C10–C16 in DI water, 0.01 M CaCl_2_, and soil extract (WG2). Additionally, CMCs of BAC-C12 and BAC-C16 were determined in three further soil extracts (OH, ISR, and AP).

### Experiments with variable ionic strength

BAC-C12 was used to test its CMC dependence on various electrolyte solutions in a likewise manner. As a measure for concentration of the electrolyte solutions of CaCl_2_, KCl, and NaCl, ionic strength was used and calculated according to

1 $$ I=\frac{1}{2}{\sum}_{i=1}^n{z}_i^2{c}_i $$,

with *c*_i_ the concentrations in mol L^−1^ of solutes *i* = 1 to *i* = n, and *z*_*i*_ the charge number for each ion.

The ionic strength of the soil extract can be approximated using an empirical relationship between ionic strength and electrical conductivity for dilute natural waters. Originally, Ponnamperuma et al. (1966) proposed a conversion constant of 0.016 in the so-called Russel equation. We used the relationship below.

2 $$ I=0.013\ \mathrm{EC} $$

In Eq. 2, *I* is the ionic strength (mol L^−1^) and EC is the electrical conductivity (mS cm^−1^) at 25 °C, based on Griffin and Jurinak (1973).

### Experiments with dissolved organic matter model compounds

For experiments with model compounds for dissolved organic matter (DOM), background solutions were prepared and employed analogously to DI water and 0.01 M CaCl_2_ solutions. Table 2 lists structural and physicochemical characteristics of the DOM model compounds employed in this study. The concentration of the DOM model compounds was chosen, so that for each compound tested, 15 mg C L^−1^ was achieved. Experiments were performed at equilibrium pH. The pH and electrical conductivity of the model compound solutions were recorded. The CMCs in the trials described under 2.3. and 2.4 were determined using the spectrofluorometric method.

**Table 2:**
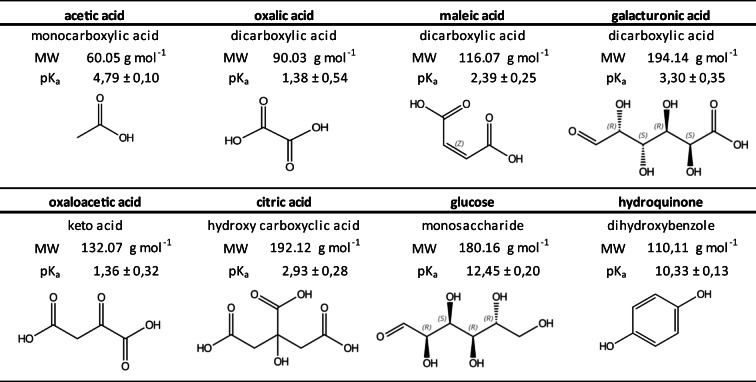
Structures and physicochemical characteristics of DOM model compounds. Data from SciFinder Scholar

### Data evaluation and analysis

Fluorescence spectra were plotted in MS Excel 2013 and values for peak ratios for the peaks at 372 and 393 nm were extracted using a procedure adapted from Dominguez et al. (1997). Raw data of all measurements are available in the supplementary information (SI).

**Fig. 1:**
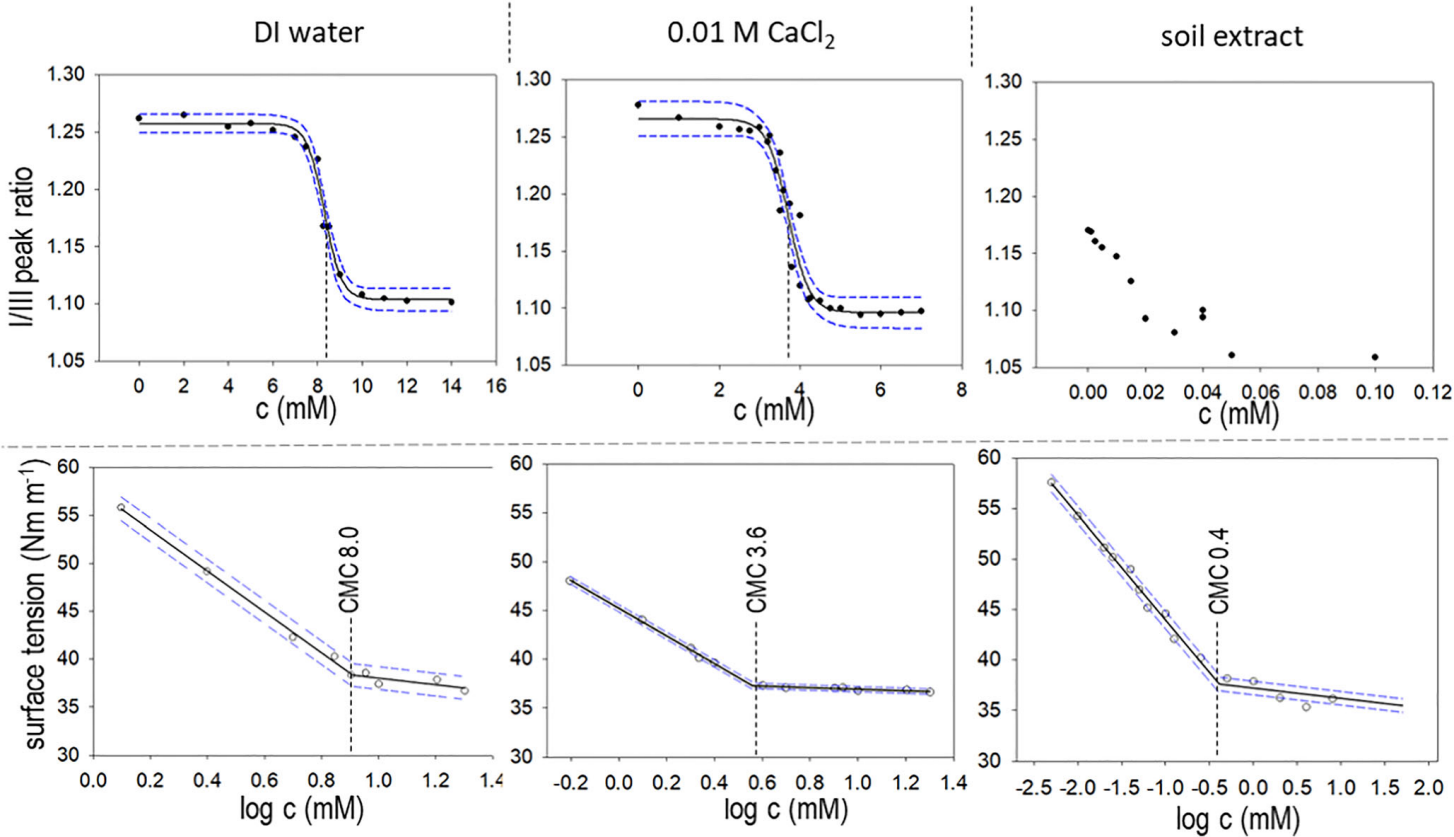
Results for BAC-C12 as an example for CMC determinations in deionized water, 0.01 M CaCl_2_, and soil extract as background medium. The upper row show data from spectroscopic determination solubilization change of sensor fluorescent molecule pyrene; the lower row shows the effect of decreasing surface tension with increasing concentration, which subsides as the CMC is reached. CMC reduction can be observed independent of method for deionized water and 0.01 M CaCl_2_. For soil solution as medium, the change in surface tension indicates the CMC more distinctively than the fluorescence sensor pyrene

Using SigmaPlot 12 (Systat Software Inc., San Jose, USA), I/III-peak-ratio data of the fluorescence measurements were evaluated in an X-Y-scatter plot. A four-parameter sigmoidal function of the general formula

3 $$ f(x)={y}_0+\frac{a}{1+{e}^{\frac{-\left(x-{x}_0\right)}{b}}} $$

was fitted to the data, with the parameter, *x*_0_, describing the inflection point of the sigmoid, and thus representing the mathematical approximation of the CMC (see example in Fig. 1). However, the sigmoidal function could not be fitted to some experimental results. In order to achieve consistent measurements, the CMCs were also determined graphically as the intercept of the fitted sigmoidal curve with the parallel in the middle of the upper and lower asymptotes, or minimal and maximum values for peak ratios. A representative example with graphical and mathematical CMC determination can be found in the SI.

Likewise, data from tensiometry measurements were plotted, this time concentration on a log scale (see example in Fig. 1, and raw data are given in the SI). Using SigmaPlot 12, a piecewise function of the general formula

4 $$ f(x)=\left\{\begin{array}{c}\frac{y_1\left({T}_1-t\right)+t-{t}_1\Big)}{T_1-{t}_1}\kern2.5em ,{t}_1\le t\le {T}_2\\ {}\frac{y_2\left({T}_2-t\right)+{y}_3\left(t-{T}_1\right)}{t_2-{T}_1}\kern1.25em ,{T}_1\le t\le {t}_2\end{array}\right. $$

was fitted to the data, with the two linear subfunctions describing the abrupt change in the dataset caused by the stagnation in surface tension decrease once the CMC is reached, so that their intersection is equal to the CMC (see also Nazrul Isalam et al. 2015). The piecewise function was suitable for most experimental results.

For all fitted curves, the 95% confidence bands, standard errors for the estimated CMCs, and regression coefficients for the fitted sigmoidal or piecewise model were calculated.

## Results and discussion

### CMCs in DI water, 0.01 M CaCl_2_, and soil extract

The shifts in pyrene peak ratios in the presence of increasing BAC concentrations clearly followed a sigmoidal shape for measurements obtained from DI water and 0.01 M CaCl_2_ for all six BAC homologs (example BAC-C12, see Fig. 1). In comparison, the surface tension plotted against increasing BAC concentrations gave a descending line, until a sharp change of slope indicated the start of micelle formation.

CMCs ranged from 0.1 to 188 mM in DI water and from 0.02 to 28 mM in 0.01 M CaCl_2_ (Table 3). Our value of 0.4/0.2 mM (fluorescence/tensiometry) for BAC-C16 falls well within the range of values reported (Table 1), which were 0.6 mM (Ismail et al. 2010), 0.004 ± 0.002 mM (Marcotte et al. 2005), 0.5 mM (Avranas and Gernátová 2007), and 0.43 mM (Itoh et al. 2005). However, Itoh et al. (2005), who also determined CMC spectrofluorometrically with pyrene as a sensor, found a value of 1.1 mM for BAC-C12, which deviates from our value of 8.3 mM. Notably, their measurement was performed after 12 h of equilibrating the BAC-C12 solution with pyrene and providing little experimental detail. Contrastingly, our measurement was performed within 5 min after the addition of pyrene, in order to minimize the effects of BAC-C12 adsorption to the surfaces of the cuvette.

**Table 3:** Critical micelle concentrations derived from fluorometric (fluor.) and tensiometric measurements (all other) for BAC homologs in three different background media. For soil extract, spectrofluorometry was not applicable, as the measurements did not yield the sigmoidal shape for CMC determination at the infliction point. Therefore, CMCs for soil extracts were measured with the Du Nüoy ring (tensiometry) method. For BAC-12 and BAC-16, CMCs were determined in extracts of four different soils. All *R*^2^ of the fitting functions were ≥ 0.9, most often ≥ 0.98. More details on the determination of CMC values and fitting parameter in SI

	DI water (fluor.)	DI water	0.01 CaCl^2^ (fluor.)	0.01 CaCl^2^	Soil extract WG	Soil extract OH	Soil extract ISR	Soil extract AP
	CMC (mM) SE	CMC (mM) SE	CMC (mM) SE	CMC (mM) SE	CMC (mM) SE	CMC (mM) SE	CMC (mM) SE	CMC (mM) SE
BAC-C8	188 ± 16	–	165 ± 11	–	–	–	–	–
BAC-C10	34 ± 1.8	36 ± 1.03	28 ± 1.2	27 ± 1.07	15*	–	–	–
BAC-C12	8.3 ± 0.2	8.0 ± 1.08	3.7 ± 0.2	3.6 ± 1.04	0.4 ± 1.2	0.7 ± 1.22	2.7*	0.11 ± 1.38
BAC-C14	1.8 ± 0.05	2.1 ± 2.1	0.5 ± 0.01	0.3 ± 1.4	n.d. ± n.d.	–	–	–
BAC-C16	0.4 ± 0.015	0.2 ± 1.14	0.05 ± 0.007	0.04 ± 1.2	0.14 ± 1.2	0.95 ± 1.59	0.06 ± 1.47	n.d ± n.d.
BAC-C18	0.1 ± 0.01	0.08 ± 1.09	0.02 ± 0.002	–	–	–	–	–

*n.d.* not detected; erratic peak ratio values; *SE* standard error at 95% confidence interval

^*^Value determined from visible flattening of curve, fit to piecewise function not sufficient

Both methods showed a clearly observable trend (Table 3) of decreasing CMCs from 118 to 0.12 mM for BAC-C8 to BAC-C18 in DI water with increasing alkyl chain length. This effect was anticipated due to the increasing hydrophobicity of the compounds. In fact, BAC-C8 with a short alkyl chain behaved quite differently from the rest of the BACs, possibly due to its predominating electrolyte rather than surfactant properties. The decrease of CMC with increasing alkyl chain length could also be reproduced with 0.01 M CaCl_2_ background electrolyte, as well as for the soil extract. Figure 1 shows a comparison of the CMC determination of BAC-C12 using spectrofluorometry and tensiometry. In both cases, the resulting CMCs determined are quite similar with 8.3, 3.7, and 0.0–0.05 mM and 8.0, 3.6, and 0.4 mM from spectrofluorometry and tensiometry measurements, respectively. A similar effect has been documented for the other quaternary ammonium compounds: alkyltrimethylammonium compounds (Asakawa et al. 2001; Akbaş and Taner 2009; Cepeda et al. 2013) and dialkyldimethylammonium compounds (Rauwel et al. 2012). For BACs, no consistent dataset existed for CMCs as a function of alkyl chain length. Remarkably, although both methods we used worked well for CMC determination in DI water and 0.01 M CaCl_2_ solution (Table 3), determinations for soil extracts with the spectrofluorometric method were hampered.

**Fig. 2:**
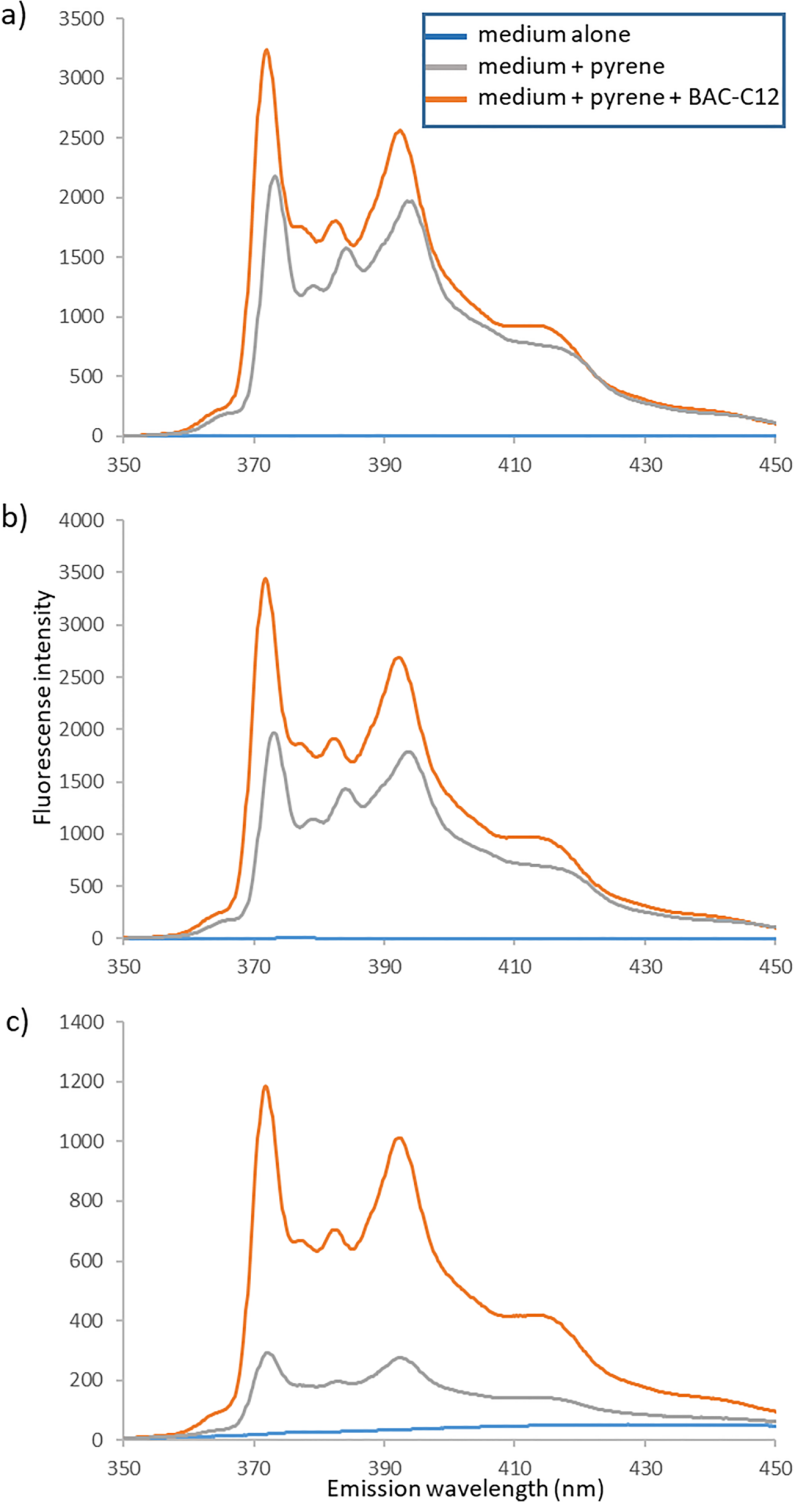
Fluorescence spectra in **a** DI water, **b** 0.01 M CaCl_2_, and **c** soil extract, as background medium. For each medium, the pyrene spectrum was measured, which also corresponded to the BAC-C12 spectra below the CMC. For the spectra medium + pyrene + BAC-C12, the concentrations were **a** 10 mM, **b** 5 mM, and **c** 0.1 mM

Figure 2 shows the fluorescence spectra of pyrene and pyrene plus BAC-C12 in different background media. For all three media, a typical spectrum was observed upon addition of pyrene with the characteristic peaks I at around 373 nm and III at 384 nm next to further fluorescence peaks. Peak IV was only barely visible as a shoulder on the blue side of peak V at 400 nm, but the spectra were identical in shape irrespective of the background medium. Overall, our data were in good agreement with published pyrene spectra (Kalyanasundaram and Thomas 1977; Dominguez et al. 1997). Also, the background medium alone did not show marked fluorescence. Although the presence of dissolved organic compounds in the soil extract was expected, the fluorescence activity of the background solution was comparatively low (Fig. 1). Thieme et al. (2016), who analyzed fluorescence excitation-emission-matrices of terrestrial dissolved organic matter, reported excitation maxima of 335 nm for a low molecular weight humic-like fraction, which would be very close to our excitation wavelength of 334 nm. However, only 30% of samples in the cited study exhibited these fluorescence characteristics. At the corresponding emission maximum at 408 nm, we did not observe such marked maximum for the aqueous soil extract.

The scale of fluorescence intensity was reduced distinctly from > 3000 in DI water and 0.01 M CaCl2 (Fig. 1 a and b) to only 1200 for soil extract (Fig. 2c). This indicates that fluorescence quenching occurred by organic compounds present in the aqueous soil extract.

For all three media, the addition of BAC-C12 altered the pyrene peaks as anticipated. A relative shift towards a smaller peak ratio occurred (Fig. 2) and this relative measure should hold true even at the reduced overall fluorescence peak intensity of the soil extract. However, the sigmoidal shape was completely lost (see example BAC-C12, Fig. 1) and data scattering made it impossible to extract a CMC value from soil extracts using spectrofluorometry.

Therefore, tensiometry should be used for soil extracts and natural waters, even if the standard error of this method was larger. For the longer alkyl chain BACs, this method yielded CMCs exceeding the values in 0.01 M CaCl_2_ (Table 3). When looking at the CMCs of BAC-C12 in the four soil extracts, they were all lower than the CMCs determined in DI water or electrolyte, but reduction varied between 3 and 70-fold that of DI water (Table 3). For BAC-C16, the CMC in 0.01 M CaCl_2_ was lower than in soil extracts and the CMC in DI water was only 1- to 3-fold reduced, maybe indicating that the counter ion effect exhibited by ions in the electrolyte was more efficient with increasing hydrophobicity of the alkyl chain. Table 4 shows an overview of the soil extract characteristic parameters, but they are difficult to relate to the CMCs observed in soil extracts, as the BAC homolog yielded variable values in the different soil extracts (Table 3).

**Table 4:** Characterization of soil extracts used as background media in CMC experiments. WP1 was used in spectrofluorescence experiment and all others were used in tensiometer experiments. WG1 and WG2 extracts were prepared from the same soil sample, but at different points in time and with different sample pretreatment (WP1: field moist, WP2: air dried)

Parameter	Unit	WG1	WG2	OH	AP	ISR
pH (CaCl_2_)	–	7.9	8.5	8.2	8.5	7.7
EC	μS cm^−1^	632	314	326	989	439
NPOC	mg L^−1^	16	41.37	64.11	417	43.72
TNb	mg L^−1^	3.4	4.6	10.2	31.7	6.16
Al	mM	0.18	0.04	0.14	0.05	n.d.
Ca	mM	1.24	1.89	1.02	3.95	1.19
Fe	mM	0.05	0.10	0.04	0.01	n.d.
K	mM	0.03	0.06	0.93	0.13	0.47
Mg	mM	0.18	0.22	0.88	3.41	0.50
Na	mM	0.10	0.17	0.20	0.07	0.65
P	mM	0.01	0.02	0.20	0.03	n.d.
S	mM	0.27	0.33	0.08	0.42	0.38

*n.d.* not detected

Overall, the tensiometric and fluorescence methods were in good agreement for CMC determination in DI water and 0.01 M CaCl_2_ (Fig. 1, Table 3). For CMC determination in soil extracts, tensiometry should be used. Curves and fitting of the piecewise fitting model showed little data scattering and the method works independent of fluorescence quenching by DOM.

### Influence of ionic strength on CMCs

**Fig. 3:**
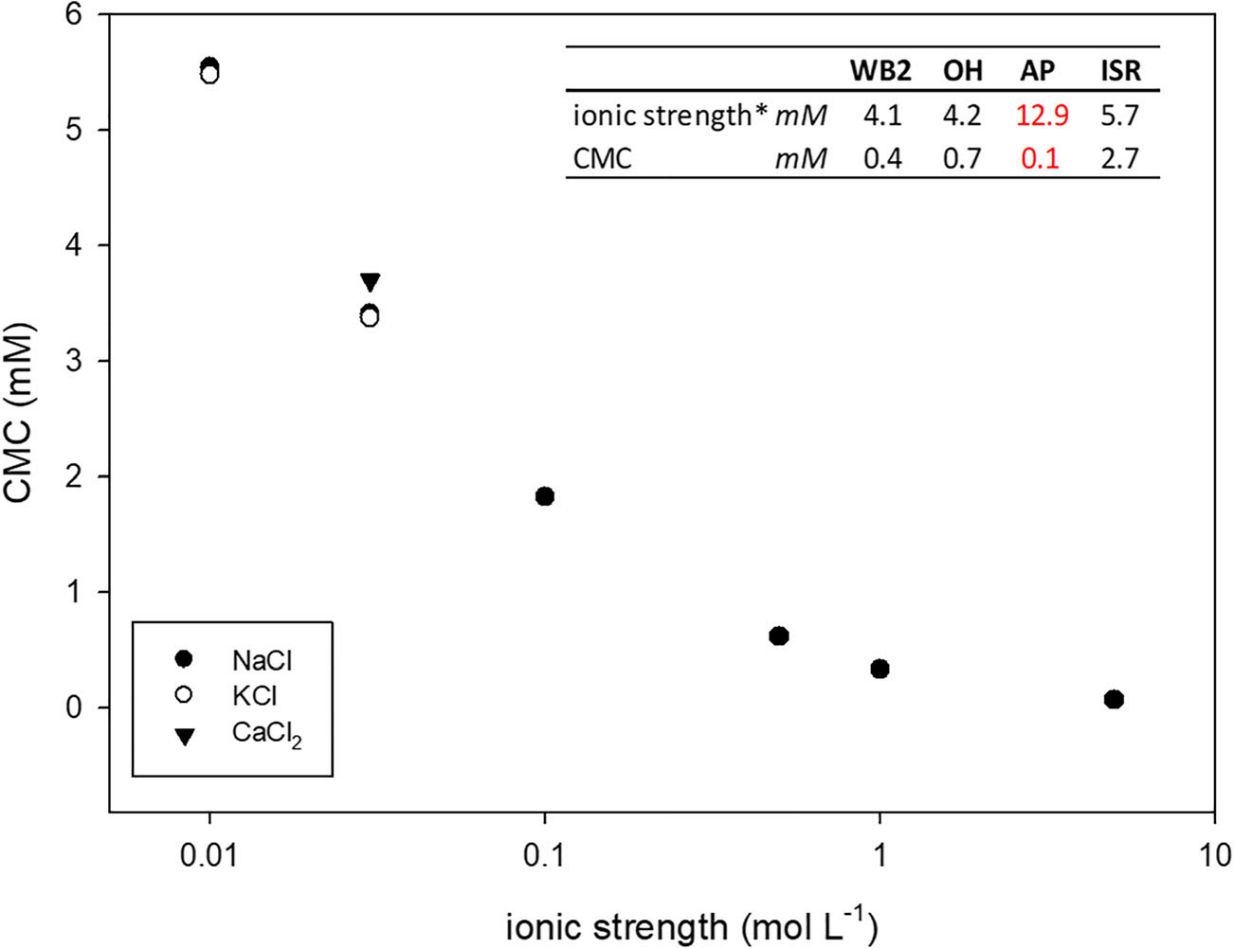
Exponential decrease of CMC of BAC-C12 with increasing ionic strength. NaCl and KCl plot congruently. Included for reference with an ionic strength of 0.03 mol L^−1^ was 0.01 M CaCl_2_ and shows only a slight deviation from the other electrolytes. Estimated ionic strength for soil extracts and corresponding CMCs of BAC-C12 were included for comparison. Asterisk indicates estimation with Griffin factor from electrical conductivity (empirical factor not valid at ionic strength > 10 mM)

The influence of ionic strength on the CMC was further investigated. Figure 3 shows the exponential decrease of CMC values with increasing ionic strength for BAC-C12, which is in agreement with effects of ionic strengths on CMC reported in the literature (Rosen 2004; Ishiguro and Koopal 2016; Aguiar et al. 2003; Brito and Vaz 1986; Dominguez et al. 1997). The increase in electrolyte concentrations in the CaCl_2_ solutions raises the polarity difference between solvent and BAC-solute, thus explaining the CMC decrease with increasing ionic strength (Table 3, Fig. 3).

The empiric relationship used for ionic strength estimation (2) is not applicable for solutions with ionic strengths exceeding 10 mM. At this point, the conductance gradually becomes proportional to the square root of concentration and linearity is lost. Due to different equivalent conductivities and proportions of major solutes in soil extracts, this relationship is only approximate. For our soil extracts, we obtained values of between 4 and 13 mM. The ionic strengths of the aqueous soil extracts were thus lower than the ionic strength of the 0.01 M CaCl_2_ solution (0.03 M). Figure 3 shows the influence of ionic strength on the CMC due to the counter ion effect with estimated ionic strength values included for reference in the small table. Based on ionic strengths of the soil extracts alone, the CMCs of the soil extracts should have ranged between the CMCs determined for DI water and 0.01 M CaCl_2_. However, the CMCs determined in the soil extracts were mostly smaller than the CMCs determined in 0.01 M CaCl_2_ and not uniformly reduced in relation to CMC in DI water (Table 3). The variability between different soils is highlighted and the applicability of tabulated CMC values for soil and sediment waters is limited, even though CMCs are characteristic properties of a surfactant (Ishiguro and Koopal 2016). The data in Fig. 3 suggested that an additional factor, other than ionic strength, also determined micelle formation in aqueous soil extracts. Further complexity was added to the interpretation of CMC reduction in soil extracts by the variation between the BAC homologs BAC-C12 and BAC-C16. Whether or not DOM composition can be an additional factor influencing CMCs of BAC homologs will be discussed in the following section.

### Influence of DOM on CMC

In addition to inorganic ions, the soil extracts contained 16 (WG1) to 400 (AP) mg L^−1^ of dissolved organic carbon (as measure of DOM), as summarized in Table 4. Out of the four soil extracts, AP with the extreme DOC concentration showed the lowest CMC for BAC-C12 (Table 3). DOM can be expected to interact with BAC molecules, thus affecting micelle formation. It is an important constituent of soil solutions, which has a crucial role in the translocation of nutrients, metals, and pollutants in soils (Kaiser and Kalbitz 2012; Seifert et al. 2016). Model DOC compounds could be various low molecular weight organic acids or larger molecules, such as polygalacturonic or tannic acids (Dultz et al. 2018). At the soil extract pH of 7.9 (Table 4), partial/deprotonated anionic charges of functional groups (e.g., carboxylic) could be attracted to positively charged nitrogen atoms in BACs and then form complexes with an altered hydrophobic nature. Additionally, the benzyl moiety of the BAC might engage in π–π interactions with phenolic moieties of DOM. Thirdly, hydrophobic interactions (i.e., dispersion interactions) between the hydrophobic tail of the BAC molecules and hydrophobic moieties of dissolved organic matter could occur.

The fact that the linear shape of the pyrene peak ratio for the BACs in soil extracts does not follow the typical sigmoidal curve (Fig. 1) was likely caused by the multitude of quenching effects of BAC-C12 and the DOM constituents. The obvious quenching of fluorescence by DOM constituents is also visible in the spectra of the aqueous soil extract in Fig. 2c. Finally, Table 5 summarizes the results from our studies with model DOC compounds at a concentration of 15 mg C L^−1^. At this conservative concentration, CMCs of BAC-C16 and BAC-C12 remained more or less unchanged in the presence of acetic and oxoacetic acid, galacturonic acid, hydroquinone, and glucose. The CMC in the presence of maleic acid even increased, but has to be interpreted with care, as its isomerization to fumaric acid would interfere with the fluorescence detection. CMC reduction in the presence of oxalic acid and citric acid was most pronounced, and this was more apparent for BAC-C16 than for BAC-C12 (Table 5). Similarly, a recent study (Ghasemi and Bagheri 2020) on mixed micelles of alkyltrimethylammonium compounds of varying alkyl chain lengths and an amphiphilic drug found that the formation of mixed micelles is increasing with increasing length of the hydrophobic side chains of the surfactant. When adjusting the pH to 6, as would be more typical for a soil solution, CMCs noticeably decreased for oxalic acid, citric acid, and galacturonic acid and also here, this effect was more pronounced for BAC-C16 than BAC-C12 (Table 5). Interestingly, when correlating the ratio of the molecular weight to the number of carboxyl groups, to the CMC of BAC-C16, a positive trend (*R*^2^ = 0.35) was observed (graph not shown). The importance of small molecule diameters and the presence of carboxyl groups could explain the efficiency in CMC reduction of oxalic acid and citric acid at pH 6.

**Table 5:** CMCs of BAC-C12 and BAC-C16 in the presence of eight different DOM model compounds at constant concentrations of 15 mg C L^−1^. Measurements were performed at equilibrium pH and EC of the solutions. For oxalic acid, citric acid, and galacturonic acid, the pH values were additionally adjusted to 6. For oxalic and citric acid, the adjustment led to a reduction of the CMC value. *R*^2^ of sigmoidal function for all measurements > 0.98 (and are not shown here)

Model substance	pH	EC (μS cm^−1^)	BAC-C12	BAC-C16
			CMC (mM) SE	CMC (mM) SE
DI water	5.8	0.6	7.75 ± 0.05	0.46 ± 0.011
Oxalic acid	3.2	233	7.76 ± 0.02	0.16 ± 0.002
Citric acid	3.8	63	8.23 ± 0.04	0.20 ± 0.004
Oxaloacetic acid	3.5	109	8.43 ± 0.05	0.42 ± 0.071
Galacturonic acid	3.9	49	8.44 ± 0.03	0.43 ± 0.003
Acetic acid	3.9	32	8.34 ± 0.02	0.45 ± 0.004
Hydroquinone	6.6	0.9	8.36 ± 0.03	0.45 ± 0.008
Glucose	5.9	0.7	8.34 ± 0.02	0.45 ± 0.004
Maleic acid	3.3	105	9.02 ± 0.09	1.09 ± 0.042
Oxalic acid pH 6	6	n.d.	7.10 ± 0.04	0.09 ± 0.002
Citric acid pH 6	6	n.d.	5.85 ± 0.02	0.05 ± 0.005
Galacturonic acid pH 6	6	n.d.	8.44 ± 0.04	0.37 ± 0.003

Mixed micelles might have quite different physicochemical properties than micelles of pure BACs, but systems studied so far were created mostly in binary surfactant systems (Tokiwa and Moriyama 1969; Cui et al. 2010). It is necessary to envision micelles not as separate phases, but rather as separate chemical species, compared with their constituting monomers (Moroi 1992). Velegol et al. (2000) documented in a material science study that sorption effects above and below the CMC differed: At concentrations greater than the CMC, the counterions of the surfactants had a stronger effect on sorption layers formed on silica surfaces, than at concentrations smaller than the CMC. Interestingly, Rauwel et al. (2012) found that nonionic surfactants inhibited the biocidal activity of quaternary ammonium compounds due to co-micellization phenomena. This decreased the monomer concentrations of the biocidal surfactants, thus resulting in a sub-inhibitory QAAC concentration present in soils to which microbial communities could develop resistance.

DOM compounds have previously been reported to exhibit surfactant-like properties themselves, so that these compounds have been found to enhance contaminant leaching from soils (Johnson and Amy 1995; Conte et al. 2005; Wu et al. 2010). It is therefore conceivable that a reduction of the CMC of QAACs might occur by micelle mixing of DOM with the surfactant.

The drop in CMC in soil solution compared with electrolyte solution that we observed (Fig. 1, Table 3) could not conclusively be explained by either the effect of electrolytes (Fig. 3) or our experiments with model compounds (Table 5). Ishiguro et al. (2007) state that the fate of DOM in waters can strongly be affected by the presence of cationic surfactants. The authors reported that flocculation of humic acid-surfactant-complexes occurred near the isoelectric point and before the CMC was reached. Inversely, the presence of polyelectrolyte substances as part of DOM might well be able to reduce the CMCs of the oppositely charged QAACs. For comparison, Taylor et al. 2003 in their surface tension measurements of an alkyltrimethylammonium compound showed that change in surface tension (as seen in our study in Fig. 1) depended on the polyelectrolyte concentration, in this case polymer polystyrene sulfonate. The authors found that critical coagulation rather than micelle formation occurs as charges are neutralized. Polyelectrolytes could thus be an interesting group of compounds for future studies.

## Conclusions

In our study, we determined CMCs of BAC homologs in DI water, electrolyte solutions of variable ionic strength and composition, and aqueous soil extracts with spectrofluorometry and tensiometry. Both methods were in good agreement for values determined in DI water and electrolyte solution, but for soil extracts, tensiometry is the preferred method, as the influence of fluorescent DOM in the extracts is avoided.

The ideal CMC in DI water determined in this study might serve as a reference point for environmental fate considerations in the absence of other useful parameters, such as log *K*_ow_ values. However, CMCs determined in DI water are of limited applicability to aqueous solutions containing additional electrolytes, such as soil solutions or sediment pore waters.

Ionic strength has a clear effect on CMC that can account for a good fraction of effects observed in soil extracts. The observed reduction of CMCs by electrolytes and DOM by partly one order of magnitude demonstrates that CMCs in soil solutions and sediment pore waters might well be reached at environmentally relevant QAAC concentrations. Differences observed between aqueous soil extracts, DI water, and 0.01 M CaCl_2_ solutions of variable ionic strength cannot be explained by the ionic strength alone.

Experiments with DOC model compounds yielded interesting first results, showing that already low concentrations of small carboxylic acids were able to reduce the CMCs of BAC-C12 and BAC-C16. The formation of mixed micelles of DOM molecules or potential interaction between polyelectrolytes and QAAC molecules could be important mechanisms driving the reduction of CMCs in natural waters, relative to DI water.

## Electronic supplementary material

ES9M 1 (XLSX 1754 kb)

ESM 2 (XLSX 203 kb)

ESM 3 (XLSX 47 kb)

ESM 4 (XLSX 184 kb)
